# Simultaneous Minimally Invasive Treatment of Colorectal Neoplasm with Synchronous Liver Metastasis

**DOI:** 10.1155/2016/9328250

**Published:** 2016-05-15

**Authors:** Stefano Garritano, Federico Selvaggi, Marcello Giuseppe Spampinato

**Affiliations:** ^1^General and Reconstructive Surgery, Department of Surgical Sciences, Policlinico Umberto I, “Sapienza” University of Rome, 00161 Rome, Italy; ^2^Division of Surgery, Casa di Cura Villa Serena, Città Sant'Angelo, 65013 Pescara, Italy; ^3^HPB and Advanced Minimally Invasive Liver Surgical Unit, Department of General and Minimally Invasive Surgery, Policlinico Abano Terme, 1 Cristoforo Colombo Square, Abano Terme, 35031 Padua, Italy

## Abstract

*Purpose*. To analyse perioperative and oncological outcomes of minimally invasive simultaneous resection of primary colorectal neoplasm with synchronous liver metastases.* Methods*. A Medline revision of the current published literature on laparoscopic and robotic-assisted combined colectomy with hepatectomy for synchronous liver metastatic colorectal neoplasm was performed until February 2015. The specific search terms were “liver metastases”, “hepatic metastases”, “colorectal”, “colon”, “rectal”, “minimally invasive”, “laparoscopy”, “robotic-assisted”, “robotic colorectal and liver resection”, “synchronous”, and “simultaneous”.* Results*. 20 clinical reports including 150 patients who underwent minimally invasive one-stage procedure were retrospectively analysed. No randomized trials were found. The approach was laparoscopic in 139 patients (92.7%) and robotic in 11 cases (7.3%). The rectum was the most resected site of primary neoplasm (52.7%) and combined liver procedure was in 89% of cases a minor liver resection. One patient (0.7%) required conversion to open surgery. The overall morbidity and mortality rate were 18% and 1.3%, respectively. The most common complication was colorectal anastomotic leakage. Data concerning oncologic outcomes were too heterogeneous in order to gather definitive results.* Conclusion*. Although no prospective randomized trials are available, one-stage minimally invasive approach seems to show advantages over conventional surgery in terms of postoperative short-term course. On the contrary, more studies are required to define the oncologic values of the minimally invasive combined treatment.

## 1. Introduction

Colorectal cancer (CRC) is the third most commonly diagnosed cancer in males and the second in females, with an estimated 1.4 million cases and 693,900 deaths that occurred in 2012 in the world [1]. The highest incidence rates are reported in Western countries with an age-standardized rate of 36.3 per 100,000 for male and 23.6 per 100,000 for female in 2012 [1]. Survival is determined by tumor stage with 5-year relative survival rates of 90.3% for Stage I and only 12.5% for stage IV [2]. The liver is the most common site of CRC metastasis, and synchronous liver metastases (SLMs) are found in up to 25% of CRC patients [3–7]. For these patients, a curative resection (R0) is the only therapeutic chance of long-term survival, although the problem of how to optimally schedule colorectal and liver operation plus neoadjuvant and/or adjuvant chemotherapy is still debated [8–12].

Different surgical strategies have been proposed to treat CRC with SLMs. Among these, the one-stage approach has been showed to be safe and effective as the classic colon-first approach, even when major hepatectomies are required [13]. This was confirmed by a recent multicenter international study that compared simultaneous versus staged approaches showing no difference in morbidity and mortality rates as well as long-term outcomes [14]. Despite opponents, the laparoscopic approach to CRC and SLMs has been demonstrated to be effective by several studies [15–20]. Recently, the one-stage minimally invasive approach (MIA) has been showed to be as safe and effective as the open treatment even for simultaneous resection of CRC with SLMs [9, 21–24]. Besides, in the last decade the use of the robotic technology has been proposed by some authors in the field of colorectal and liver surgery as an alternative to laparoscopy, in order to overcome some technical limitations [25–27]. The aim of this review is to analyse current literature concerning one-stage MIA for CRC and SMLs patients, with particular emphasis on technical issues and perioperative and oncologic outcomes.

## 2. Methods

### 2.1. Inclusion and Exclusion Criteria

We analysed recent published studies that describe the clinical course of patients who underwent one-stage resection of CRC with SLMs totally by MIA, including laparoscopic or robotic-assisted procedures. Case reports and case series were retrospectively reviewed. An intention to treat analysis was applied and, therefore, cases converted to open procedures were included. On the contrary, cases treated with combined laparoscopy/laparotomy procedure (hybrid technique) or hand-assisted procedure were excluded. In addition, clinical conditions in which minimally invasive results are not clearly reported or not distinguished from conventional surgical outcomes were excluded. The Brisbane 2000 Terminology of Liver Anatomy and Resections was used to define minor or major liver resection [34]. According to this, anatomic and nonanatomic hepatic parenchymal resections, even in association with radiofrequency ablation (RFA), were included.

### 2.2. Medline Research Criteria

A systematic search was conducted using Medline (through PubMed) for all reports published until February 2015 (the last search was performed on February 28th). The search words for the literature review were scheduled in four groups:

- *First group*: “liver metastases” and “hepatic metastases”.
- *Second group*: “colorectal”, “colon”, and “rectal”.
- *Third group*: “minimally invasive”, “laparoscopy”, “robotic-assisted”, and “robotic colorectal and liver resection”.
- *Fourth group*: “synchronous” and “simultaneous”.

The search terms were designed by combining one word from each group, so that all possible combinations were employed. This process yielded 96 search terms, all of which were sought in titles and/or abstracts of English written papers. Results were enriched by 32 additional articles, manually searched or listed in the reference. Finally, two authors (Stefano Garritano and Federico Selvaggi) reviewed all abstracts independently and the full text of relevant studies was considered for inclusion.

## 3. Results

**Figure 1:**
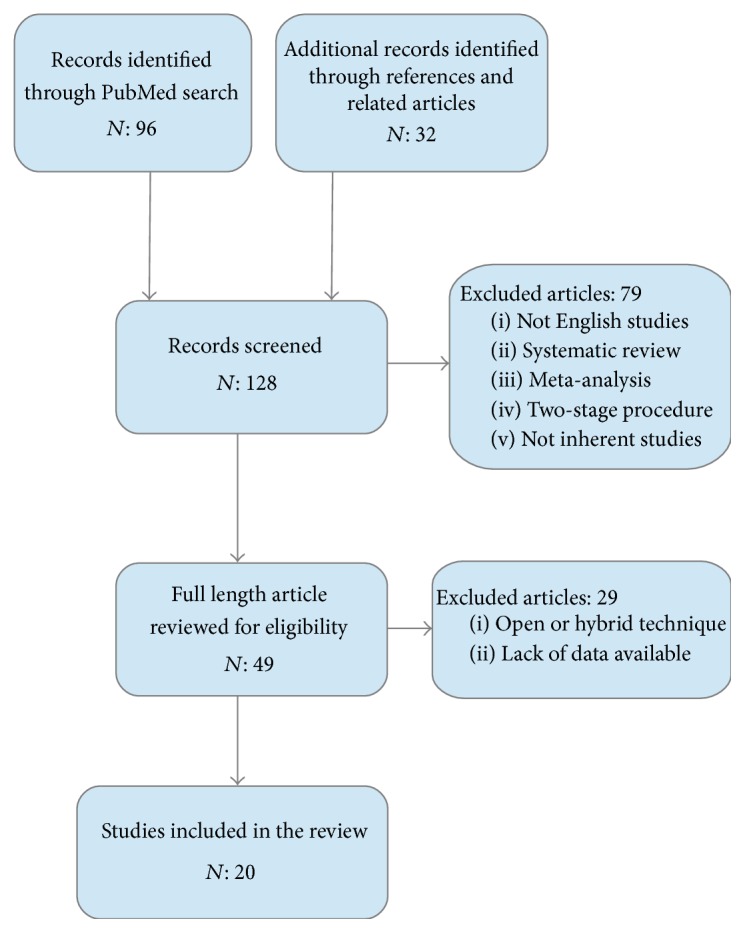
Flow chart showing selection of articles.

The research was brought to a total of 128 manuscripts of which only 49 were suitable for inclusion in the study. Of these, 29 papers were excluded according to the reported criteria, and finally a total of 20 papers were analysed (Figure 1). No randomized clinical trials were found. We assessed retrospective clinical data of 150 patients who underwent one-step MIA for primary CRC with SLMs.

### 3.1. Patients and Neoplasm Characteristics

Demographic and clinic-pathological data are reported in Table 1. Median age of patients was 60 years (range, 31–88). According to American Society of Anesthesiologists (ASA) grade, the patient health status was classified as ASA I-II in 52 (34.7%) cases, ASA III-IV in 23 (15.3%) cases, and unknown in 75 (50%) of cases. CRC was diagnosed in right colon in 35 (23.3%) cases, left colon in 36 (24%) cases, and rectum in 79 (52.7%). SLMs were single in 81 (54%) patients, multiple in 49 (32.7%), and unknown in 20 (13.3%) cases. Location of SLMs was unilobar in 131 (87.3%) and bilobar in 19 (12.7%), respectively. Eleven (7.3%) patients underwent neoadjuvant chemotherapy (Table 2).

### 3.2. Operative Outcomes

Operative outcomes are shown in Tables 2 and 3. All procedures but one (0.7%) were completed by MIA with no conversion to hybrid or open procedures. The approach was laparoscopic in 139 (92.7%) cases and robotic-assisted in 11 (7.3%) cases. According to CRC location, there were 35 (23.3%) right colectomies, 36 (24%) left colectomies/sigmoidectomies, 68 (45.3%) anterior rectal resections (ARR), 10 (6.7%) Miles procedures, and one (0.7%) subtotal proctocolectomy. Temporary ileostomy was reported in 8 (5.3%) patients who underwent ARR. Liver resection was minor in 134 (89.3%) patients and major in 16 (10.7%) cases, respectively. Liver resection was nonanatomical in 90 (60%) cases and anatomical in 60 (40%) cases. The first performed procedure was liver resection in 56 (37%) cases and colorectal in 84 (56%), while it was unknown in 10 (7%) cases. Intermittent Pringle's Manoeuvre (IPM) was frequently prepared but finally used only in selected and limited conditions. Total median operative time was 320 minutes (range, 120–749 minutes). Median estimated blood loss was 259 mL (range, 10–1500 mL) (Table 3).

### 3.3. Perioperative Outcomes

Overall morbidity and mortality rates were 18% and 1.3%, respectively (Table 4). There was one patient who experienced uncontrolled bleeding that finally required conversion to open surgery. Postoperative complications are reported in Table 4. The most common complication was colorectal anastomotic leakage (3.3%). Median length of hospital stay was 8.5 days (range, 3–54 days). Perioperative mortality within 30 days was reported in 2 (1.3%) cases (Table 4).

### 3.4. Oncologic Short-Term Outcomes

Only 14 authors have reported oncologic outcomes with recurrences in a cohort of 107 patients. Preoperative CRC diagnosis was confirmed by histology in 147 cases (data not included in the table). The SLMs diagnosis was not confirmed postoperatively in 5 cases as reported by two authors [10, 22]. There were 101 (67.3%) cases of R0 status and 6 (4%) cases of R1 and 43 (28.7%) were the unknown status.

## 4. Discussion

Although the optimal strategy for resectable CRC with SLMs has not been established yet, the one-stage approach for simultaneous colectomy and hepatectomy gives the advantages to avoid two surgical procedures thus reducing risk for patient and costs for the community while keeping acceptable morbidity and good oncologic results [13, 35]. Recently, MIA for simultaneous resection of CRC and SLMs has become popular [10, 29, 31, 33, 36]. Modern combined MIA, by laparoscopic and/or with robotic assistance, although being in its preliminary phase of experiences, has been showed to be feasible and safe even in cases requiring major liver resections [21, 25, 30, 36]. In the present study perioperative outcomes of 150 patients, affected by stage IV CRC and SLMs treated by MIA up to February 2015, were retrospectively analysed. The reported data confirmed the feasibility and safety of simultaneous MIA with acceptable perioperative morbidity and mortality. Surprisingly, despite the high rate of ARR the number of temporary ileostomies was low [6, 10, 22, 28]. This could be explained by the high rate of minor liver resections and the apparently infrequent use of IPM to perform it. In fact, prolonged vascular clamping is responsible for transient portal hypertension with oedema of the intestinal mucosa that ultimately might be leading to colorectal anastomotic failure [21, 37]. In this review, only 16 patients underwent minimally invasive colorectal resection associated with a major hepatectomy, confirming that this type of procedure is performed only by few specialized centers. In addition, most of the cases, including major and anatomical liver resection, were performed by two different specialized teams allowing good results in terms of conversion and perioperative outcomes. In fact, the morbidity and mortality rate were similar to the conventional open approach [32, 38]. In more than 50% of cases, colorectal resection was the first procedure performed. Instead, as other authors, we believe that the choice of carrying out the liver resection as first step of treatment gives to the surgeon the opportunity to change surgical strategy from a combined procedure to a “liver first” resection which has been showed to be another effective treatment for stage IV CRC [39]. Indeed, this happened in one patient of our series, who showed intraoperatively chemotherapy related steatohepatitis, that finally developed transient life-threading postoperative acute liver insufficiency [21]. In this review only five patients experienced colorectal anastomotic failure but we could not understand if this complication was associated with type of operation performed at first, or time of vascular clamping, prolonged operative time, and blood loss. Interestingly, only eleven patients underwent neoadjuvant chemotherapy prior to surgical therapy. The limited use of neoadjuvant chemotherapy along with a limited number of performed major hepatectomies could explain the overall low rate of complications found in this review. In fact, it is well known that chemotherapy associated steatohepatitis and volume of resected liver are associated with an increased number of post-liver-resection complications, including bleeding and liver failure [40]. Unfortunately, data regarding overall survival and recurrence were too heterogeneous to understand whether or not preoperative chemotherapy affected the oncologic results in terms of overall survival and recurrences. Other limits of this retrospective analysis were the difficulties in resuming data concerning the microscopic diffusion of the disease, such as distance of resection margins, number of harvested lymph nodes, and tumor stage classification. These criticisms reflect the nonhomogeneity of reported data, concerning R0 status or local and distal recurrence of the disease. However, two retrospectives studies have reported no differences in terms of overall survival, while a faster surgical recovery was observed for MIA patients [9, 32].

In order to overcome the limitations of laparoscopy, some authors have advocated the use of the da Vinci® surgical system for liver resection [41, 42]. Wristed instruments offering seven degrees of freedom, tremor filtration with stereoscopic steady view, together with optimal working ergonomics, and avoidance of “fulcrum effect” are the main strengths of the robotic platform. No doubt exists about the fact that these technological improvements enhance surgical dexterity [43]. Recently, some authors have demonstrated that an increased number of patients can receive a laparoscopic major hepatectomy, especially when robotic assistance was used [41, 44]. However, in our review the majority of cases was performed laparoscopically and the use of the robotic platform was limited. These data might reflect the infancy of the robotic technology and the related costs of the procedure [45].

## 5. Conclusions

One-stage MIA for CRC patients with SLMs can be performed safely with an acceptable morbidity. Reduced hospital stay and the faster enrolment of patients to adjuvant treatments represent the most relevant advantages of MIA compared with conventional surgery. Indeed, MIA offers all the benefits of laparoscopic minimally invasiveness, especially in terms of fewer postoperative adhesions. This represents a crucial aspect when redo liver surgery is indicated for recurrences. With all the criticisms of a retrospective and noncomparative analysis, this review showed that MIA for simultaneous colectomy and hepatectomy can be performed safely even in cases requiring major liver resections, when the combined procedures are performed by specialized and well trained teams. Prospective and randomized trials are needed to define the oncological benefits and validate the role of one-stage MIA for CRC patients with SLMs.

## Figures and Tables

**Table 1:** Demographic and clinical characteristics.

Variable	Patients n. 150 (%)
Age (years), range	60 (31–88)
ASA (n.)	
Unknown	75 (50%)
ASA 1-2	52 (34.7%)
ASA 3-4	23 (15.3%)
Location of primary tumor (n.)	
Right colon	35 (23.3%)
Left colon	36 (24%)
Rectum	79 (52.7%)
Number of liver metastases (n.)	
Unknown	20 (13.3%)
Single	81 (54%)
Multiple	49 (32.7%)
Location of SLMs (n.)	
Unilobar	131 (87.3%)
Bilobar	19 (12.7%)
Size of SLMs (n.)	
Unknown	85 (56.6%)
≤2 cm	22 (14.6%)
>2 cm	43 (28.8%)

*ASA:* American Society of Anesthesiologists classification and *SLMs:* synchronous liver metastases.

**Table 2:** Surgical procedures for primary tumor and perioperative treatment.

Authors	Clinical study	Number of patients	Right colectomy	Left colectomy	Rectal resection	Miles	Minor hepatectomy	Major hepatectomy	Neoadjuvant CHT/RT	Adjuvant CHT/RT
Leung et al. [28]	CR	1	—	—	1 (1 ileostomy)	—	1	—	—	1
Choi et al. [27]	CR	1	—	—	1	—	1	—	—	—
Sasaki et al. [29]	CS	9	2	—	5^*∗*^	2	9	—	—	—
Patriti et al. [26]	CS	6 (of 7)	1	4	1	—	6	—	1 CHT 1 RT	—
Casaccia et al. [20]	CR	1	—	—	1	—	—	1	—	1
Lee et al. [6]	CS	10	4	—	6 (2 ileostomies)	—	9	1	—	—
Lupinacci et al. [3]	CR	1	—	1	—	—	1	—	—	1
Tranchart et al. [30]	CR	2	1	1	—	—	—	2	1	—
Polignano et al. [31]	CS	13 (of 20)	7	2	4	—	13	—	—	—
Hoekstra et al. [7]	CR	2 (of 5)	1	1	—	—	2	—	—	—
Hu et al. [32]	CM	13	3	4	1	5	11	2	—	12
Spampinato et al. [21]	CS	4	—	3	1	—	—	4	4	4
Ida et al. [24]	CS	10	2	3	4	1	10	—	2	—
Aljiffry et al. [33]	CR	1	—	—	1	—	1	—	1 CHT/RT	—
Inoue et al. [23]	CS	8 (of 10)	2	3	3	—	8	—	1 CHT	6
Lin et al. [9]	CM	7	—	4	2	1	7	—	1	7
Ando et al. [18]	CR	2	—	1	1	—	2	—	—	—
Jung et al. [22]	CM	24	2	1	21 (3 ileostomies)	—	18	6	—	—
Liu et al. [19]	CR	1	—	—	1	—	—	1	—	—
Berti et al. [10]	CS	34 (of 35)	10	8	15 (2 ileostomies)	1	34	—	—	34
		*Tot. 150*	*Tot. 35*	*Tot. 36*	*Tot. 69*	*Tot. 10*	*Tot. 133*	*Tot. 17*	*Tot. 12*	*Tot.66*

*CR:* case report, *CS:* case series, *CM:* case matched, *CHT:* chemotherapy, and *RT:* radiotherapy.

^**∗**^4 rectal resections and 1 subtotal proctocolectomy.

**Table 3:** Surgical outcomes.

Surgical procedure (n.)	
Totally laparoscopic	139 (92.7%)
Laparoscopic, robotic-assisted	11 (7.3%)
First step procedure (n.)	
Unknown	10 (7%)
Liver approach	56 (37%)
Colorectal approach	84 (56%)
Operation for primary tumor (n.)	
Right colectomy	35 (23.3%)
Left colectomy	36 (24%)
Anterior rectal resection	68 (45.3%)
Miles procedure	10 (6.7%)
Subtotal proctocolectomy	1 (0.7%)
Temporary ileostomy (n.)	8 (5.3%)
Hepatectomy (n.)	
Minor resection (<3 segments)	134 (89.3%)
Major resection (≥3 segments)	16 (10.7%)
Anatomical resection	60 (40%)
Nonanatomical resection	90 (60%)
Conversion to laparotomy (n.)	1 (0.7%)
Intermittent Pringle's Manoeuvre (n.)	10 (6.7%)
Operative time (min)	320 (range 120–749)
Estimated blood loss (mL)	259 (range 10–1500)

**Table 4:** Postoperative outcomes.

Overall morbidity (n.)	27 (18%)
Postoperative medical complications (n.)	13 (8.6%)
Thrombocytopenia	1
Postoperative ileus	3
Myocardial infarction	2
Pleural effusion	2
Pneumonia	1
MOF	1
CVC infection	1
Ictus cerebri	1
Deep vein thrombosis	1
Postoperative surgical complications (n.)	14 (9.3%)
Primary anastomotic leakage	5
Bile leakage	3
Liver abscess	2
Colovaginal fistula	1
Postoperative intestinal obstruction	1
Site infection	1
Unknown	1
Hospital stay (days), range	8.5 (range 3–54)
30-day mortality (n.)	2 (1.3%)

## Abbreviations

CRC: Colorectal cancer
SLMs: Synchronous liver metastases
MIA: Minimally invasive approach
IPM: Intermittent Pringle's Manoeuvre.
